# Performing in the Absence of Debilitating Anxiety: A Qualitative Analysis from the Perspective of Professional Western Classical Ensemble Musicians

**DOI:** 10.3390/bs16060896

**Published:** 2026-06-02

**Authors:** Thomas J. Nicholl, Maree J. Abbott

**Affiliations:** Faculty of Science, School of Psychology, The University of Sydney, Sydney, NSW 2050, Australia; maree.abbott@sydney.edu.au

**Keywords:** performance anxiety, music performance anxiety, cognitive models, professional musicians, values-driven performance

## Abstract

Performance-related anxiety for musicians, or Music Performance Anxiety (MPA), is often considered to be ‘part of the gig’. Whilst research has been conducted to elucidate the experiences of musicians who perform with anxiety, research on the experiences of those who perform in its absence is scarce. Learning from these experiences can support the development of treatment interventions and performance-related experiential goals for musicians, especially for those who are yet to perform without experiencing impairing anxiety. The aim of this study was to examine the cognitive and schematic experiences of professional musicians who perform in the absence of debilitating MPA using qualitative interview methods. Six professional western classical ensemble musicians were recruited (three female; three male) to complete a semi-structured interview examining the cognitive and schematic features of their performance experience. A thematic analysis was completed, describing six core themes across three layers comprising early experiences, pre-performance and during-performance experiences. Participants identified that despite early career experiences of MPA, they developed confidence through repetition and the positive influence of others. They relied upon foundational technical, performance and mental skills to perform, which encompassed a strong answer to the question of ‘why’ when it came to performing. During performances, participants reported a deep sense of connection to the music, their peers, and the audience, externally focusing their attention beyond themselves. The results highlight the utility of interventions aligned with the experiences of those who perform in the absence of debilitating anxiety and call for continued values-driven cognitive performance preparation education for musicians.

## 1. Introduction

### 1.1. Overview

Research investigating high-performance pursuits has substantially grown in recent decades, with institutions, organisations, and individual teams searching for the ‘edge’ when it comes to performance. One area of particular interest across performance domains is the management of performance-related anxiety, which is no less prevalent for professional musicians (Kenny, 2011). Situated in the *Diagnostic and Statistical Manual of Mental Disorders—Fifth Edition* (DSM-5-TR; American Psychiatric Association, 2022), Performance anxiety (PA) acts as a specifier for social anxiety disorder (SAD). PA encapsulates the performer’s experience of intense anxiety when faced with a real or perceived audience, where performers will often persist with their performance, despite experiencing intense fear and anxiety (Powell, 2004). PA is widely recognised as a reality for musicians, with prevalence rates of music performance anxiety (MPA) estimated to be between 16.5% and 60% (Fernholz et al., 2019). Some musicians may never have experienced a performance in the absence of anxiety or have a clear idea or goal regarding what such a performance would be like. PA is often classified as a subjective experience, reliant on one’s interpretation of their environment, physiological state, and capabilities. This facilitative–debilitative dichotomy has most recently been investigated by Murphy et al. (2024), exploring the role of MPA for musicians, as well as in clinical studies of PA in the context of social anxiety (e.g., Thomas & Johnco, 2025). Murphy et al. (2024) found that a large subgroup of musicians reported that anxiety was facilitative, and that appraising anxiety in this way was also associated with significantly greater levels of positive emotion and less MPA than those appraising anxiety as debilitating. These findings highlight the potential for subjective appraisals of performance-related anxiety to influence whether MPA is experienced as debilitating. Recent reviews have highlighted the normal and adaptive role of anxiety in the context of high-pressure and high-standard musical performances, as well as the role of unhelpful cognitive, attentional, emotional, and behavioural factors that contribute to the experience of debilitating MPA (Herman & Clark, 2023; Nicholl & Abbott, 2024).

Current research has identified that individuals who report PA (more commonly known as MPA in the context of music performance) experience more cognitive symptoms regarding their performance (Fuentes-Rodriguez et al., 2018). Dominant models of PA in the anxiety literature more broadly highlight the relationship between maladaptive core-beliefs, associated assumptions, and negative automatic thoughts, resulting in anxiety and avoidance and the use of safety behaviours that contribute to the development and maintenance of anxiety (Barlow, 2002; D. B. Clark & Agras, 1991; D. M. Clark & Wells, 1995; Heimberg et al., 2014a, 2014b, Hofmann, 2007; Rapee & Heimberg, 1997). Musicians often persist in these negative performance experiences, marked by apprehension and anxiety, despite having the skills and talent to perform (Powell, 2004). More specifically, frameworks for SAD/PA emphasise the relationship between initial performance apprehension and pre-performance rumination, resulting in increased negative self-focused attention (as opposed to other/task-focused attention), which is further impacted by negative perceptions of self, low perceived skill, high social cost, and anticipation of failure, resulting in the development of safety/avoidance behaviours and post-event anxiety (Barlow, 2002; D. M. Clark & Wells, 1995; Hofmann, 2007; Rapee & Heimberg, 1997).

Facilitating and debilitating experiences of MPA have been explored, with research also investigating the adaptive experiences of performers (Herman & Clark, 2023). Given the strong cognitive features of MPA (Osborne & Franklin, 2002), understanding the cognitive and schematic features of those who perform with and without anxiety is relevant to inform treatment interventions and anticipated experiences for performers. It is also relevant for individuals and clinicians aiming to identify clear intervention targets, informed by the potential experience of performance in the absence of debilitating MPA (Nicholl & Abbott, 2024). Prior to the development of these goals, it is important to better understand the experiences of those musicians who perform without substantial anxiety.

### 1.2. Qualitative Experiences of Musicians

Musicians experience a range of debilitating symptoms, including negative cognitive and ruminative processes (Kenny, 2011, 2023), strong physical sensations such as panic, trembling and nausea (Steptoe & Fidler, 1987), and negative performance experiences, often resulting in maladaptive coping strategies (Wesner et al., 1990). Recent studies have used qualitative methods to understand musicians’ experience of MPA from both a debilitating perspective and a coping perspective (Huang & Song, 2021; Huang & Yu, 2022; Kenny, 2011; Tahirbegi, 2022). This research also integrates the impacts of experiences outside of the individual and contributing factors such as organisational, cultural, and systemic influences.

Kenny (2011) documented the experiences of musicians who performed with a range of anxiety levels, and importantly, explored the role of external factors that influence the experience of musicians. Kenny (2011) found that early music mentors/teachers/conductors often negatively influenced the development of anxiety, often through fear-based practices, which were maintained through poor management at an organisational level. Kenny (2011) documents several examples, including the negative impact of a conductor’s criticism through public humiliation in rehearsals and through the development of biases and impressions of other musicians. More recent literature has challenged the necessity of these practices given their detrimental effect on musicians, whilst also acknowledging that evaluation is an embedded component of the industry (Herman & Clark, 2023). It is also acknowledged that performances are at least somewhat inherently threatening, with common adages of “you’re only as good as your last performance” ringing true when opportunities are based on subjective performance-based experiences (Herman & Clark, 2023).

Robinson and Nigbur (2018) built on the findings of Kenny (2011), noting the relevance of the musician’s identity and sense of self as key themes that substantially contributed to the negative and debilitating experiences of some professional musicians. A strong sense of self was found to come from performing, including one’s attachment to performing well, with musicians placing greater importance (and pressure) on themselves to perform well to affirm their sense of self and self-esteem. Often, these strong attachments to success and performing well stemmed from influential parental relationships and could often be understood from a generational perspective (Kenny, 2011). Robinson and Nigbur (2018) went further, noting the importance of trust and the impact of the audience in contributing to the sense of self and musical identify felt by undergraduate music students. The audience plays a pivotal role in influencing musicians’ appraisal among these students, with limited trust for the audience further influencing musician’s attachment to their performance.

Papageorgi and Welch (2020) identified themes surrounding the intensity of the musician’s experience (both physically and psychologically) while performing and the impact of this on the quality of their playing (both positive and negative), the development of coping strategies, and whether they are ‘achieving release’ from their experiences. The results highlighted the range of negative physical symptoms, attempts to ‘control’ these symptoms, the drive to flee, concerns about letting people down or others identifying your experience (through a change in performance/musical quality), and a focus on problem-based strategies (i.e., more practice!). Pleasingly, performers found reprieve from this experience by having a supportive community and being pragmatic in their reflections about their experiences. The severity of symptoms during performances and the depth of emotional experiences for musicians demonstrates that the experience is felt beyond the performance, with preparation and post-event anxiety occurring for many musicians.

### 1.3. Adaptive Performance Experiences

Few studies have specifically investigated the experiences of non-anxious performers and how their experience can inform performance-based goals for musicians experiencing heightened and severe anxiety. T. Clark et al. (2007) identified musician’s overall perceptions and experience of performance, examining the value in preparation and pre-performance routines, musician’s thoughts/perceptions about themselves and their environment, and stressors faced by musicians and how they combat them. Musicians reported strong values, using their performance to express and challenge themselves, and often relying on adequate preparation and remaining relaxed in anticipation of their performance. Often, musicians equated the level of success of the performance to their level of preparation. These results demonstrate performance-based preparation and emerging values-based identification as important to performance experiences.

Emerging research in the music performance field has looked at the role of enhancing performances, rather than solely focusing on anxiety reduction. Often, this explored the role of facilitative arousal (Murphy et al., 2024), which is distinguished from anxiety that can negatively impact the performance experience. Other avenues of research have assessed the relationship between task complexity and arousal level using the Yerkes–Dodson curve (Yerkes & Dodson, 1908), and its relationship with the level of task mastery and practice (Kenny, 2011). Developments such as the Individual Zone of Optimal Functioning (Hanin, 2000) have been examined in pianists, highlighting their value for individuals in identifying arousal levels that support their individual performances (Yao & Li, 2022). Similarly, some interventions for MPA target skill-building as opposed to symptom reduction, such as mental skills-based programmes, show emerging positive results (Hoffman & Hanrahan, 2012; Steyn et al., 2016).

More recent research in the music performance field has investigated the role of flow in capturing and influencing musician’s experiences of MPA (Antonini Philippe et al., 2022; Cohen & Bodner, 2019b; Spahn et al., 2021). Spahn et al. (2021) investigated the relationship between flow and MPA in musicians, demonstrating the positive impacts of flow on functional coping and in reducing symptoms of MPA. Spahn et al. (2021) found that professional musicians are more likely to experience flow when compared to non-professional musicians, and that older musicians were likely to experience more flow, in addition to the individual importance of the performance, further enhancing the flow experience. These findings demonstrate the characteristics that can lead to a performance that is not dominated by MPA, leading to a more favourable experience for musicians. Cohen and Bodner (2019b) similarly explored the relationship between MPA in musicians and flow, with results showing that classical musicians regularly experience flow in their performances, in addition to finding an inverse relationship between MPA and flow, further supporting the findings of Spahn et al. (2021) and Fullagar et al. (2013).

Antonini Philippe et al. (2022) recently assessed the qualitative positive experiences of performers regarding the development of a flow state when performing. Key themes emerged around social standing, performance preparation, connection to one’s body, awareness of skills and self-confidence, intrinsic motivation, attentional focus, and transcendence. Whilst MPA was not assessed or the core focus of the study, the findings demonstrate that, even if anxiety is present, there are several factors which support the development of positive performance experiences. Antonini Philippe et al. (2022) suggested that these factors could act as mediators for the impacts of anxiety and stress on performance, with mental/psychological preparation as a core recommendation for supporting musicians in developing effective coping and performance skills. Objective-setting, cognitive restructuring, bodily awareness, and attentional control were highlighted as strategies to promote the development of performance practice, and perhaps in turn manage the harmful impacts of stress and anxiety on musicians.

Beyond flow, Yao and Li (2022) applied the Individual Zones Optimal Functioning (IZOF) model to college pianists, assessing whether its application could improve their subjective performances. Following the identification of an IZOF for each of the participants (based on retrospective performances), participants prepared for an evaluated performance, completing anxiety measures before their performance. Following their performance, their initial IZOF, pre-performance anxiety scores, and performance evaluations were collated. The results demonstrated that anxiety scores for most participants fell within their IZOF and most received positive evaluations. These findings highlighted the importance of individual differences in anxiety for musicians and their differing impact on performance, especially in the context of levels of arousal and MPA (see also Murphy et al., 2024).

These studies demonstrate factors that promote alternate experiences of MPA, which may be helpful in refining the performance experience and goals of musicians experiencing substantial MPA. Understanding the specific cognitive and schematic experiences from the perspective of the processes identified in cognitive models of SAD may further identify relevant specific targets for interventions to both reduce MPA (reactive targets) and increase positive performance experiences (proactive targets).

### 1.4. Aims and Hypothesis

Research continues to emerge highlighting the experiences of musicians who perform. In recognising the documented experiences of musicians who perform with debilitating anxiety, and the experiences of those who perform with facilitative and perhaps optimal levels of arousal, this study aims to further explore the experiences of professional musicians who perform in the absence of debilitating anxiety. The experiences of those who perform with the facilitative lens of arousal does not necessarily indicate the experience of performing in the absence of substantive anxiety. To explore this experience, we sought to investigate the perspective of musicians who subjectively do not experience debilitating MPA in relation to the processes identified in cognitive models of those who experience MPA, including attentional focus, state/trait anxiety, rumination, performance and threat appraisal, and avoidance/safety behaviours. Applying the processes identified in dominant cognitive models of anxiety to musicians performing without debilitating anxiety could further inform treatment interventions by providing a better understanding of how these musicians ameliorate the impact of the currently identified maintaining factors.

As identified by Antonini Philippe et al. (2022), mental/psychological preparation and skill development is necessary to support musicians in developing valued careers. Extending on the cognitive factors that influence positive performances (i.e., attentional control), explicitly exploring the cognitive and schematic features of professional musicians who perform in the absence of debilitating anxiety could help uncover the mechanisms employed by these individuals that allow them to perform free of debilitating anxiety. Learning from this sample will aid recommendations for the approaches that professional performers take, and determine whether these insights can be applied to younger performers or other performers experiencing difficulty managing anxiety.

The aim of this study is therefore to explore the cognitive and schematic features of musicians who perform in the absence of anxiety, specifically from the perspective of disorder-specific cognitive processes, such as those identified by Hofmann (2007). It is anticipated that professional musicians will have well-rehearsed mental skills and conceptualisations of their performances which foster a healthy level of arousal. It is hypothesised that the dominant themes identified will support disorder-specific cognitive models of SAD (Hofmann, 2007), whereby individuals are able to manage any anticipatory feelings of anxiety, appropriately (re)direct their attention away from their internal experience and toward the task at hand, have a healthy and realistic concept of their ability and the audiences’ expectations, reappraise their internal experience of anxiety as helpful to facilitating their performance, and perform in the absence of safety or avoidance behaviours.

## 2. Method

### 2.1. Design

To explore the cognitive and schematic features of musicians who perform in the absence of debilitating anxiety, professional musicians were invited to participate in a semi-structured interview to explore their current cognitive practices, beliefs, and behaviours around performance. Interview questions were developed in line with Hofmann’s (2007) cognitive behavioural model of SAD, focusing on baseline anxiety (trait anxiety), pre-performance anticipation (pre-event rumination), attentional focus during performances (threat appraisal and self-focused attention), and post-performance processes (post-event rumination, avoidance, and safety behaviours during performances). The questions developed to guide the semi-structured interview are listed below. Question 1 (Q1) assessed early experiences of MPA, asking participants to recall their earliest and previous experiences and how they managed MPA at these times. Questions 2 and 6 (Q2, Q6) asked about anticipatory and post-performance experiences, and Questions 3 to 5 (Q3 to Q5) explored musician’s experiences during performances.

Q1. Have you ever experienced music performance anxiety? If so, how did you manage it? To what extent were those strategies successful?
Q2. What is your attitude toward performing? How do you feel about performing (before, during and after)?
Q3. What do you do when you perform? Where is your attention directed?
Q4. Who are you performing for? What is your perception of the audience?
Q5. What do you think others expect of you during your performances? Do you think you can meet those expectations? Do you think you have different expectations/goals?
Q6. What do you think about post-performance? Do you elicit feedback?

### 2.2. Sample

We chose to interview professional musicians to assess the experiences of those who had achieved excellence technically and professionally in their field, for whom performance was a mandatory component to maintain employment. To be eligible for the study, participants needed to identify as earning most of their income through performing music, be over the age of 18 years of age, be an Australian resident, and be fluent in English. Participants who previously experienced performance anxiety but who did not currently experience substantial anxiety in relation to performance were considered to meet the inclusion criteria for participation. Participants were not screened for current methods of reducing MPA (such as using medications or substances, or accessing interventions). It was assumed as part of the recruitment process that musicians were not currently experiencing MPA and that they did not use medication for this purpose. Significant efforts to recruit participants were made, with the hopes of interviewing a range of professional musicians across genres (classical, jazz, orchestral, solo, tutti, etc.).

### 2.3. Participants

Participants (*N* = 6) responded to a research flyer distributed by the authors to various musical organisations and institutions. Due to the specific population we assessed, only music-relevant demographic information was obtained to further protect privacy and anonymity. There was an equal number of male (*n* = 3) and female (*n* = 3) participants, with a range of classical musical instrument specialties (cello, viola, violin, voice, and clarinet). Years playing ranged from 25 to 53 years (*M* = 37.2; *SD* = 9.2). All participants scored below the clinical cut-off of 11 on the M-MPAS (Mazzarolo & Schubert, 2021) indicating low levels of self-reported performance anxiety. Participating musicians all performed as tutti (ensemble) Western classical musicians in professional setting. All musicians identified performing as a soloist in some capacity; however, when interviewed they were relying on ensemble work. This group of musicians (western classical) are hypothesised to more typically experience higher levels of MPA than those working in other musical genres (Papageorgi et al., 2011), with classical musician’s experiences of anxiety commonly being reported (Casanova et al., 2025). The sample size presented is comparable to qualitative studies by Antonini Philippe et al. (2022), T. Clark et al. (2007), and Papageorgi and Welch (2020).

### 2.4. Measures

*Demographics*—Participants were asked demographic questions to confirm their age range, number of years playing, primary instrument, any secondary instruments, performance type (solo, ensemble, mixed), highest level of training, and employment other than performing music.

*Performance Anxiety*—Level of performance anxiety was measured using the Mazzarolo Music Performance Anxiety Scale (M-MPAS; Mazzarolo & Schubert, 2021). The M-MPAS was developed as a brief five-item scale to assess the extent of performance anxiety for musicians. Items assess frequency, intensity, and avoidance tendencies are measured using a 7-point Likert scale ranging from ‘strongly disagree’ (0) to ‘strongly agree’ (6). Items in the M-MPAS include: ‘I experience strong nerves/anxiety before I perform’; ‘I frequently experience nerves/anxiety before I perform’; ‘I avoid performing in order to alleviate my nerves/anxiety’, ‘I feel positive before my music performances; and ‘I don’t want to go ahead with my performances because of my nerves/anxiety’. Scores on the M-MPAS range from 0 to 30, with a score of 11 or above considered the clinical cut-off for performance anxiety (Mazzarolo & Schubert, 2021). The M-MPAS has demonstrated sound internal reliability (α = 0.89) and has also shown a strong and positive correlation (*r* = 0.80) with Kenny’s (2009) Music Performance Anxiety Scale, Revised (K-MPAI-R). The Cronbach’s alpha for the M-MPAS in the present study was adequate (α = 0.65).

### 2.5. Procedure

The study was approved by The University of Sydney Human Research Ethics Committee [2022/721] and informed consent was obtained prior to completing the study. Participants were directed to a Qualtrics survey to assess their eligibility when responding to the flyer for the present study. After consenting to the study, participants completed a series of demographic questions prior to completing an MPA measure (M-MPAS; Mazzarolo & Schubert, 2021) to assess their self-reported level of performance anxiety. All participants who signed up to the study were eligible and were interviewed and did not indicate significant levels of MPA on the M-MPAS. Participants were contacted to arrange a time for the 60 min semi-structured interview, which was conducted via Zoom with the first author and recorded for transcription purposes. An interview-based research protocol was developed that included questions exploring participants’ experience of performing in the absence of substantial anxiety.

### 2.6. Data Analysis

Given the emerging evidence surrounding the absence of performance anxiety in musicians, a Thematic Analysis (TA) methodology was considered most appropriate for analysis of the interviews. The six core stages of TA were followed, in line with the protocols set out by Braun and Clarke (2006). The recorded Zoom interviews were transcribed by the first author. The data was then coded by the first author, before general themes were developed. Key themes were reviewed, and sub-themes emerged, which were then named and defined. The overarching themes were then analysed in line with the previous literature and presented in relation to the aims of the study (Braun & Clarke, 2006). A doctoral-level clinical psychologist acted as the second rater and reviewed all interview transcripts, endorsing all themes with no new or additional themes suggested. The second author was also in agreement regarding the final themes.

A deductive approach was taken to the data, given the influence of past research based on cognitive models of SAD and the emerging experiences of musicians who perform with substantial anxiety. We sought to explore predominantly pragmatic approaches to music performance, whilst also touching on the phenomenological experiences of the musicians interviewed. Given the specificity of the sample size, and the wealth of research exploring MPA in individuals to date, ‘information power’ was used to determine when recruitment ceased, as opposed to ‘saturation’ (Malterud et al., 2016).

Information power refers to the value of the information gathered from qualitative interviews, as opposed the saturation of data following a larger number of interviews. The notion of information power is based on five key tenants: the study aim, its specificity, the theory available, the dialogue of the interviews, and analysis. In cases where studies have a narrow aim and a dense research area, and are driven by applied theory, a strong dialogue occurs between the interviewer and participant, and the analysis is considered on a case level (rather than on a cross-case level). In the case of the present study, we aimed to specifically target a minority population group (professional musicians in Australia who do not suffer from MPA); as such, the specificity of the experiences identified is likely to be dense (participants work in an industry with limited employment opportunities), the quality of the dialogue is likely to be strong (the interviewer conducting the study has worked extensively within both the fields of music and psychology), and the analysis strategy is focused on case-specific information (rather than comparing across cases).

This approach also aligns with the work of Braun and Clarke (2021), who argued for the inclusion of pragmatism when establishing a criterion for when to cease data collection, recognising the value of the data as it is, in addition to the challenge of lengthy recruitment periods. In making these decisions, we also acknowledge that the experience of those in the performance space is varied and interwoven with their internal experiences. Moreover, an action-focused axiological approach was also taken, where epistemological value is placed on the transferability of ideas that ontologically suggest the knowledge is context-specific (Creamer, 2018).

## 3. Results

The results are presented in keeping with the overall structure of the semi-structured interviews, with participants naturally reflecting on their performance careers as they explored early music experiences, pre-performance experiences, and performance experiences. Quotes from participants were included to elevate the quality of the text and as exemplars of the themes that emerged. Participants’ responses were deidentified, with codes used throughout the text indicating responses for musicians (M) 1 to 6 (M1, M2, M3, M4, M5, and M6).

**Figure 1:**
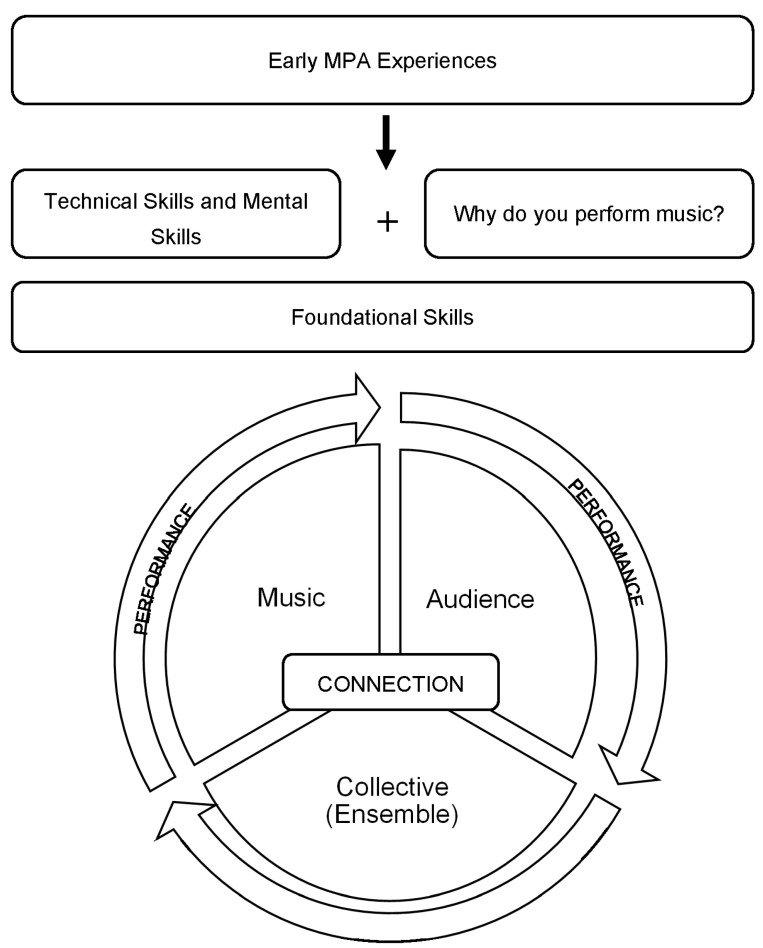
Relationship between key themes.

Key codes and subthemes appeared repeatedly across participant responses. As summarised in Table 1, participants demonstrated consistent early experiences and conceptualisations of anxiety as a taboo topic, leaning on early MPA experiences and repeated exposure to performance environments to prime their performance experience. Participants reported relying on their core foundational skills, with technical, performance, and mental skills demonstrated. These core foundations allowed musicians to connect deeply to the music and their performances, aided by their colleagues, who were seen as part of their collective (ensemble), and the audience, who were viewed as allies. Table 1 depicts the core themes linked with each layer, and Figure 1 provides a visual depiction of the relationship between each theme.

### 3.1. Early Experiences of Music Performance Anxiety

Participants unashamedly recalled early experiences of MPA. They each described early adolescence as a time when anxiety was at its highest, with auditions and early exposure to performances being particularly provoking. For some, this extended into the early phase of their professional career and often persisted until they settled into consistent performance routines. The anxiety experienced was described as mild to moderate, with none identifying debilitating anxiety that required intervention. Although trait levels of anxiety were not discussed, no participant described experiencing significant levels of anxiety externally to their music careers.

*I experienced [MPA] at university, there was a time where I wasn’t performing constantly and during auditions…my heartbeat was right up and I almost [felt like] I was having palpitations.*

*(M1)*

Participants also reflected on the impact of employment opportunities and career considerations on their experiences of performance anxiety.

*I was terrible with auditions and terrible with competitions. That’s when I would particularly feel performance anxiety, and then I think I had the insight that for me it was I mean, there’s always the self-critic, the self-judge is there and the doubt about your capacity to perform, and that would go into overdrive, depending on the assessment I made of the audience who was listening to me…*

*(M2)*

*Yes, I did… there will be times when I would be affected by it. The expectations got higher. So, in a professional sphere there was pressure from employment to make sure I did the right thing, otherwise you’d be out of the job, or they wouldn’t rehire you…*

*(M3)*

This was also impacted by the experiences of playing more exposed roles, such as solos, or experiences where ‘the stakes were high’.

*My first time playing the piece. And I played the solo, and I just my breathing was getting heavy…so I got to the end, but I was having to breathe like every bar, because my breathing was so bad, and then, like I went to the hotel that night was just like I could barely move because of all the tension.*

*(M5)*

*I definitely have [experienced anxiety], and I would say, as I have gotten older, it’s gotten better. I think the stakes that you know it was a job, or it was an audition… are really high, and at that time… you think… not life or death, but it’s really important to you to do well, and the higher the stakes are, of course, the more the anxiety goes up.*

*(M4)*

Most were not able to directly identify how or when their anxiety began to reduce, but almost all attributed this to an increase in confidence through repeated performance experiences: “[It got to a stage] where I wasn’t learning a new repertoire every week and I became more comfortable” (M5); “There is a sense of excitement, which I think comes after a sense of calm and of preparation” (M3); “So, on a very practical level, performing a lot definitely helps” (M4). This then became something that participants identified as helping to manage anxiety as they could fall back on their technical and performance experience.

*You fall back on, you know, your standard performance. You’ve made that at a certain level, so that whatever happens, if you have to fall back on that… and that’s kind of where I set my expectations of myself.*

*(M2)*

Many of the participants spoke of the prevalence of MPA throughout their workplaces: “[it’s] like this dirty little secret that no one talks about…you know, some people literally have to take Beta Blockers every day to do their job like that is the reality…” (M5). Such anxiety was described to be enhanced by historical ‘fear-mongering’ tactics used by organisational leaders, with participants saying “you don’t wanna be yelled at in front of 80 people like as an adult or as a child” (M5); “conductors sometimes have a way of talking to people as if you’re in the naughty corner, and then better conductors have a way of just talking about the music and just approaching it from that. We’re sort of all in it together on a punitive kind of thing” (M4).

### 3.2. Foundational Skills

When exploring how participants were able to overcome any anxiety they felt, they often described two key components: (1) early exposure to performance experiences, and (2) the reliability of foundational skills. These skills often included the development of base technical skills related to their instrument. One participant highlighted the utility of returning to ‘your sound’:

*I go back to the very basic concepts…break things down, think before you play, reiterate how important your sound is, you will feel it confident…whether the passage is all technical stuff no one cares about, they’re going to enjoy it as long as you sound great…then you can do anything!*

*(M5)*

Similarly, the idea of just “being prepared and being comfortable…going back to your structure” (M3) was highlighted. One of the benefits to being prepared is that performers had the ability to be flexible, “you learn everything you need to do your work…and then you’re free to be who you want to be in the moment…you can do anything you want in the moment…you’ve got to learn it, get it right, and then forget it” (M3). At the top level, there is also the assumption that you’ve done something right, so your “standard performance is [something] you can fall back on” (M2). The benefit of being prepared then means “you know what to expect, you know you can do it…and there’s no reason to be anxious” (M6).

Participants expanded on preparation and exposure to performance as key foundational components and discussed their use of flexible psychological skills that they could use in preparation for their performances and their performance life in general. Such skills surrounded an emerging mindset towards performance, where many acknowledged a balanced perspective on the role music has in society, “You’re just playing …you’re not saving lives…it’s a dose of reality” (M5). Many also spoke of the ability to shift their attention from themselves to their environment. For example, M1 spoke of a particular technique that helped them detach from ‘big moments’ and shift their attention away from their instrument. Skills such as optimism, curiosity, and flexibility also featured in many conversations, with a strong movement away from listening to an internal self-critic and a move toward being compassionate regarding one’s performances:

*I’m certainly not beating myself up…my satisfaction is not about anyone else…I think I’m happy with who I am in this part of my life, rather than endlessly seeking more and more and more to fill an empty hole in myself…you’ve got to be your own best friend.*

*(M3)*

### 3.3. Why Do You Perform Music?

Foundational skills were often accompanied by a strong sense of understanding why they chose to be performing musicians. Without prompt, each musician depicted a foundational philosophy that was almost used to override any anxiety they experienced. This ‘why’ was unique from person to person and was often associated with their own personal reasons for pursuing music. M6 spoke about their love, passion, and curiosity for music and music-making. They identified that music is a ‘gift’ and spoke of it as an opportunity to contribute.

*I saw the value in it… I’m very curious about music, so I’m always listening …and feeling inspired and stimulated by it…and that feeds into the mindset of what I’m doing and why I’m doing it….the basic idea that there is great music here and it’s a treasure for everyone, there’s a legacy …it’s a gift for everyone to be inspired by…I feel a very strong sense of wanting to share that with people.*

*(M6)*

M3 spoke more generally about their love for music and how it “helps” them. This links directly to their preparation and purpose behind their performing:

*I generally do love music…I love what I do, and that really helps…so going out on stage and doing a concert is not some sort of ordeal…it’s not a difficult issue…I’m looking forward do it…It’s the love of it, it’s fun!*

*(M3)*

M1 and M5 equally spoke about performing for the love and joy of music, but also spoken of the opportunity to perform for others (the audience): “People just literally love us…they’re our biggest fans”.

### 3.4. Connection to the Music

When asked ‘what happens when you are performing?’ each musician alluded to a strong sense of connection to the music. An almost ethereal connection led each individual to describe an externally focused link between themselves and what they were producing. M5: “You’re just in the moment, you’re noticing”.

*Just be in the moment…if you make a mistake, you feel more resilient when you’re in the moment…you can respond more appropriately to it…in the moment, you’ve prepared well, it’s the foundation…it’s the moment where art is made.*

*(M3)*

Many expanded further on their externally focused attention: “you’re not fully focused on yourself, because it’s not all about you…you’re a part of a team” (M5); “It’s mechanical [at times] …if I’m conscious of myself it’s sort of uncomfortable… but when you’re in it…in the moment…you’re free to do what you want in the moment!” (M3); “it’s not all about me…if I’m really self-focused it seems to make everything worse…I listen equally to myself and to other people, I think that works to take the pressure off [and] I end up playing better” (M4); “you’re collaborating with other people, it’s a conversation…I’ll focus on the music or on what’s going on around me or in the audience out there…not really myself” (M6).

This was often referred to as their own ‘flow state’, where the individual was able to activate a sense of timelessness and deep connection to themselves and the performance environment.

*It’s the best performances [where] you get into a flow state and it’s just happening. You’re certain. If things are stressful, you’re not in flow state…you ground yourself, and your awareness opens up and widens, and then you can reactive your flow state.*

*(M2)*

### 3.5. Performance as a Collective (Ensemble) Effort

When asked about where their attention was focused, each musician romantically described an unspoken relationship with the music made by their peers. Interestingly, the impact of peers and the connection they felt emphasised the collective or ensemble role of the group in ensuring the success of a performance. A sense of camaraderie was not necessarily described, rather a sense of collective shared performance. This was particularly poignant in this group, as all participants held strong ensemble roles that relied on a ‘collective’ performance.

*We’re all in it together…one of the greatest things is a sense of ensemble…so when everyone is on the same page, the power of what we’re doing is…putting it all together so that we’re all one and in a sense its sort of a miracle moment…we’re creating a world in front of people. *

*(M3)*

*If I’m self-focused that tends to make everything worse. I don’t really care about how I’m playing, but if I listen equally to myself and to other people, I think that works to take the pressure off…and I end up playing better. You’re always working with colleagues who you really respect and admire, and there’s always something fantastic to listen to…it’s not just all about me!*

*(M4)*

This extended to their connectedness to the music and how it could benefit the overall performance.

*So, I’m playing a bar, but who am I giving it to? Or where is it coming from? Who am I playing with here? That expands your listening…and you get out of your own bubble and become part of a bigger painting…it’s not about you, your part of a team.*

*(M5)*

*You think about the importance of listening to everyone else…you must listen…collaborating with other people…it’s a conversation with other people, and you have to be engaged with what they’re doing.*

*(M6)*

### 3.6. The Audience Is Your Ally

The audience was described as an active participant in the performance space. Interestingly, participants described an almost communal relationship. Here, the audience can provide the musicians with positive feedback, without necessarily detracting from the musician’s experience. Many spoke about the excitement that surrounded performing with audiences, “the whole kind of feeling around the concern, people are excited and dressed up”, and how such excitement is fed back to the musicians as a perceived expectation that they “enjoy themselves [while performing]” (M5). The audience’s excitement is then linked to gratitude for their presence:

*I love playing my instrument…I like playing with other people…but I love playing for others [the audience] …the best part of it is the community aspect and making a living from moving people.*

*(M1).*

*[I feel] gratitude! I’m glad that they’re there; if they weren’t, I wouldn’t have a job! It’s also a gratitude to know I’m not the only person that enjoys music and you know the whole idea of a shared experience…it adds a dimension to just doing it on your own…you feel a sense of doing something in a community kind of minded way, I’m doing it for other people, I’m doing it with other people….and it’s very satisfying.*

*(M6)*

Even the idea of the audience knowing whether a mistake was made was discussed, with one musician alluding to the fact the audience is not as familiar with the performance as they were; therefore, if you made a mistake they would not realise: “The audience won’t know… they’ll just enjoy what you do with it! “(M3).

In this context, the audience as an ally is perhaps a more appropriate term than the audience as a dependant. One participant made sure to highlight that “you can’t rely on the audience to dictate your mental state” (M2). This point was associated with the foundational skills used by musicians, and particularly their ‘why’. Not one participant identified ‘performing for the audience’ as their sole reason for performing, or ‘performing for accolades and fame’. This also highlights the importance of connection to the piece, and of the externally focused attention described by each of the musicians.

## 4. Discussion

The aim of this study was to explore the experiences of professional musicians who perform in the absence of anxiety, with qualitative interviews informed by disorder-specific cognitive models of social anxiety disorder (e.g., Hofmann, 2007). We hypothesised that the experiences of professional musicians who perform in the absence of anxiety would directly contrast with the factors hypothesised to maintain performance anxiety. That is, we anticipated that professional musicians peforming without debilitating anxiety would adaptivly view the expectations of their impending performances as something they could meet, leading to a degree of positive anticipation toward the performance. Moreover, we anticipated that during the performance, musicians would focus on the connection they had to the music and their ability to meet the perceived expectations of the audience, and that they therefore would not experience subsequent rumination, nor seek to modify their preparation or performance in order to achieve particular outcomes.

The scope of our findings is limited to the population who participated in the study; even then, the results likely only describe the experiences of a select few. Following efforts to recruit, six experienced, professional, Western classical ensemble musicians were interviewed to explore their current performance experiences. Each musician confirmed that they do not currently experience MPA in the context of relying on performing as a musician to make most of their income. Whilst levels of training, primary instrument and job experience varied, all of the musicians identified as experiencing MPA in early adolescence. Over time, they developed a set of coping skills to manage their anxiety in order to continue performing, ultimately without debilitating levels of MPA.

The results of this study uncovered the adaptive, curious, and deeply connected experiences that these musicians have to their perfomances. The findings provide some evidence of the ubiquitous exeriences of anxiety and the adaptive processes one can develop to not only curb anxiety but foster a valued union between the musician and their craft. The insights gained support the notion that performance skills and perspectives can be taught alongside music performances, and the lived expierences of those who thrive in performance spaces are invaulable in understanding and helping those who suffer.

These findings appear to align with previous research on flow and the positive experiences of performances (Antonini Philippe et al., 2022; Cohen & Bodner, 2019a, 2019b, 2021; Csikszentmihalyi, 1990; Fullagar et al., 2013; Kirchner et al., 2008; Robb & Davies, 2015; Spahn et al., 2021). Participants directly commented on their own experiences of flow, whilst also indicating their cognitive skills to externally direct their attention to the music, their peers, and their overall experience. In line with the findings of Antonini Philippe et al. (2022), participants used value-guided action and a sense of ‘higher-self’ to direct their performances, perhaps further enhancing their externally driven attention away from possible internal experiences of anxiety. Interestingly, the experience of connection to their audience, colleagues, and the music could be interpreted as a complete, deep immersive experience (i.e., flow).

The findings also largely support the inverse use of Hofmann’s (2007) disorder-specific model of SAD in accounts of how participants described managing MPA. Hofmann’s (2007) model suggests that in the lead up to and during performance experiences, negative performance appraisals, negative attentional focus and biassed cognitive processes are activated that result in catastrophic misinterpretations of the performance environment and one’s ability, facilitating subsequent avoidance behaviours and rumination, and maintaining anxiety. The accounts from participants highlight that MPA is not necessarily something you can ‘dodge’; rather, it is an experience that can be managed through the development of skills/techniques and a shift in perspective from unhelpful thoughts and a negative attentional focus toward performance and collaboration with fellow musicians and the audience.

Nonetheless, some of the participants described cognitions that do not align with more modern approaches to understanding anxiety within performance environments. The idea that “there’s no reason to be anxious” is contrary to recent explorations of the role that MPA plays in music circles. Herman and Clark (2023) describe the role MPA plays in not only the classical musical field, but the pedagogical approach of some administrators. Similar to applications in sport psychology, developing acceptance-based approaches to anxiety highlight the role of understanding one’s relationship with anxiety and its inevitability, appearing during things that are important to the individual (such as performing music in front of others). In keeping with this perspective, Clarke et al. (2020) demonstrated the effectiveness of acceptance-based interventions in building psychological flexibility and reducing MPA, despite the naturally occurring anxiety in response to music performance situations. A recent review details the promise of acceptance-based interventions for musicians in professional and educational contexts (Juncos & de Paiva e Pona, 2018). A musician can be competent at performing without feeling confident in their abilities. This may be reflective of the changing tone and attitude toward MPA in some musical circles.

This shift in perspective is particularly beneficial in light of the role of ‘anxious apprehension’ in anxiety disorders (Barlow, 2002). Whilst participants were not able to directly identify any causal relationship linked to decreasing levels of MPA, a shift in attitude and perspective regarding their performances may have been a key component. The rise in the popularity of the term ‘mindset’ (another term for attitude or perspective) has led to the distinction of various dichotomies used to classify a set of beliefs regarding a task, i.e., growth vs. a fixed mindset (Dweck, 2017), opportunity vs. threat (Krueger & Dickson, 1993), and abundance vs. scarcity (Freebairn-Smith, 2010).

These constructs have also been translated to SAD research more broadly, with the exploration of a ‘shyness mindset’ as a positive belief that social inhibition will allow for skill acquisition, development, and overall social curiosity (Valentiner et al., 2011). Here, beliefs about shyness are suggested to predict levels of impairment of SAD over time (Valentiner et al., 2011). Whilst studies are yet to explicitly measure the relationship between MPA and a particular mindset, there is growing recognition of the benefits of cognitive reappraisal, modifying beliefs about worry (Borkovec et al., 1999) and rumination (Wong & Moulds, 2010), and building a more adaptive attitude toward anxiety and performance.

Managing and modifying attitudes to performance may be the key to stemming and overcoming the development, exacerbation, and maintenance of MPA. The findings support the use of cognitive models, such as Hofmann’s (2007) model, to begin to train musicians (of any stage) in preventative (and restorative) ways of managing MPA. This is focused on recognising the relationship between anticipatory thoughts in the lead-up to a performance, the role of shifting attentional focus (i.e., outwardly, toward the music, the audience, and your colleagues), one’s interpretation of external cues in the performance context (e.g., audience response), and how one thinks about performances afterwards (e.g., absence or management of negative post-event rumination). This is not dissimilar to the applicability of the cognitive–behavioural models identified by Nicholl and Abbott (2025), as well as the recent finding from a systematic review and meta-analysis that cognitive–behavioural treatments are the most studied for individuals with MPA (Nicholl & Abbott, 2025). Examples of research, such as that of Osborne et al. (2007, 2014), demonstrate the value of cognitive–behavioural-based psychological skills training interventions, where anxiety is reframed as an adaptive performance-enhancing experience.

Early intervention and skills groups might focus on the development of an adaptive mindset that utilises anxiety adaptively to perform. This could be as early as music classes, when children start to learn their instrument and become exposed to musical performances. This might, however, go further, looking at embedding mental skills and psychological skills training into secondary and tertiary musical performance studies. This suggestion has already been highlighted by Antonini Philippe et al. (2022), who suggested that mental/psychological skills trainings focusing on the cognitive factors related to MPA and positive performance experiences can be taught/trained to minimise the impact of MPA on the performances (and overall careers) of musicians.

Music teachers and institutions would benefit from proactive involvement in promoting a culture for musicians that focuses on developing skills to identify and manage the MPA experienced by students (MacAfee & Comeau, 2023). Promoting teaching and an institutional culture that explores attitudes towards performance, and which reframes the audience and fellow performers as allies, would be beneficial. More recent approaches to treatment have begun to explore the role of including teachers in interventions (Shaw et al., 2020), in addition to individual skills-based approaches (Cohen & Bodner, 2019a; Hoffman & Hanrahan, 2012; Steyn et al., 2016).

### 4.1. Limitations

Despite lengthy efforts to recruit (online, word-of-mouth, flyers), only a small sample of musicians participated. This resulted in a very focused group of experienced ensemble musicians, who appeared to come from similar performance backgrounds. This study did not focus on other components which may impact levels of MPA, such as performing predominantly as soloists, whether musicians perform with and without music, and whether they also perform in other roles (such as acting, being on stage, or performing in a pit). Feedback from organisations and institutions highlighted a reluctance to explore MPA in their setting, reporting concerns that exploring MPA may exacerbate symptoms. This provided some helpful qualitative information around the attitude towards MPA, indicating that future studies recruiting musicians need to account for systemic attitudes towards MPA. Whilst a small sample size is often a limitation of qualitative research (Braun & Clarke, 2021), the utility of the information power within these results helps to reduce this limitation (Malterud et al., 2016). The sample is, however, limited in its application to other genres and experiences of other performers. Western classical performers occupy a large proportion of ageing musicians, with younger performers not necessarily taking traditional routes in training and performance compared to their predecessors. Similarly, it is difficult to generalise the experience of six Western classical musicians to the experiences of all performers in this genre. Future studies would be wise to replicate the research questions of this study to examine the adaptive experiences of musicians, collecting more performance details to ascertain if there is a particular characteristic supportive of lower levels of reported MPA. The fact that MPA is more widely researched and discussed in current times may naturally reduce its levels within younger populations, and it is therefore important to examine a wider breadth of experiences, characteristics and genres among musicians. Replication of this study is necessary to broaden the generalisability of these results, especially with different levels of performance experience (e.g., university students) and performance types (e.g., soloists). Comparisons of the experiences of classical and non-classical professional musicians are also warranted. Future qualitative studies could also explore the trajectory of musicians’ experiences over a particular period of their careers, looking to map their levels of anxiety and attitudes towards performances as they mature.

### 4.2. Conclusions

The experiences of the musicians who candidly participated in this study clearly demonstrates the joy and connection music can bring, and the role that attitudes towards performance can play in ameliorating unhelpful beliefs about our role in successful performances. Performances are opportunities to demonstrate skill and share in the joy of music-making with both peers and the audience, in which long and fulfilling careers are possible. The results of this study encourage further assessment of the cognitive and attentional factors that contribute to MPA and support the early teaching of skills to musicians at all stages to help manage the detrimental impact of MPA.

## Figures and Tables

**Table 1:** Thematic Structure.

	Theme	Code
Early Experiences	Early experiences of MPA	Early experiences Confidence through repetition Influence of others (mentors/conductors)
Foundational Skills	Music performance skills	Building technical skills Mental skills
	“Why do you perform music?”	Enjoyment Value Vocation
Music Performance	Music performance is a collective (ensemble) effort	Passing the music on Performing as a group Anxiety stemming from peers
	Connection to the music	Flow Externally focused attention Focus on production, not processes
	Audience as allies	Performing for the audience “They’ll never know” Feedback

## Data Availability

Due to ethical obligations in protecting participant anonymity, supporting data is not available to be shared.
